# Sex Differences in the Impact of Metabolic Dysfunction-associated Fatty Liver Disease on the of Patients with Hepatocellular Carcinoma After Radical Resection

**DOI:** 10.7150/jca.83779

**Published:** 2023-04-17

**Authors:** Junzhang Huang, Suosu Wei, Yuntian Tang, Qiuhuan Zhang, Honglin Luo, Zhenyong Tang, Yi Tang, Hongjun Liu, Wei Huang, Xiaofeng Dong, Jianrong Yang

**Affiliations:** 1 Department of Hepatobiliary, Pancreas and Spleen Surgery, Guangxi Academy of Medical Sciences, People's Hospital of Guangxi Zhuang Autonomous Region, Nanning 530021, Guangxi, China.; 2 Department of Scientific Cooperation of Guangxi Academy of Medical Sciences, People' s Hospital of Guangxi Zhuang Autonomous Region, Nanning 530021, Guangxi, China.; 3 Department of Colorectal and Anal Surgery, The People's Hospital of Guangxi Zhuang Autonomous Region & Guangxi Academy of Medical Sciences, Guangxi, China.; 4 Institute of Oncology, Guangxi Academy of Medical Sciences, the People's Hospital of Guangxi Zhuang Autonomous Region, Nanning, Guangxi, People's Republic of China.; 5 Department of Hepatobiliary, Pancreas and Spleen Surgery, Guangxi Academy of Medical Sciences, People's Hospital of Guangxi Zhuang Autonomous Region, No.6 Taoyuan Road, Qingxiu District, Nanning 530021, Guangxi, China.

**Keywords:** Hepatocellular carcinoma, Metabolic syndrome, Radical resection, Sex

## Abstract

**Background:** International experts have put forward a new definition for metabolic dysfunction-associated fatty liver disease (MAFLD). Nonetheless, sex differences in MAFLD function in hepatocellular carcinoma (HCC) survival is still unknown. Therefore, the current work focused on exploring the gender-specific association of MAFLD effect on prognosis after radical resection of liver cancer.

**Methods:** The long-term prognostic outcomes of 642 HCC patients undergoing hepatectomy were analyzed retrospectively. To calculate overall survival (OS) and recurrence-free survival (RFS), Kaplan-Meier (KM) curve was plotted. Further, using Cox proportional model to explore the prognostic factors. Sensitivity analysis was performed using propensity score matching (PSM) to balance the confounding bias.

**Results:** For MAFLD patients, median OS and RFS times were 6.8 years and 6.1 years, respectively, compared to 8.5 years and 2.9 years in non-MAFLD patients. KM curve shown that compare with non-MAFLD patients, MAFLD patients had a higher survival rate in men, but had a lower survival rate in women (*P*<0.05). Multivariate analysis showed that MAFLD was significantly risk factor with mortality in the female (HR = 5.177, 95%CI: 1.475-18.193). However, MAFLD was not related to RFS This correlation was consistent after PSM analysis.

**Conclusions:** MAFLD can improve the mortality of women undergoing radical resection for liver cancer, which independently estimate disease prognosis but is not related to recurrence-free survival.

## Introduction

Hepatocellular carcinoma (HCC) ranks third among cancer-associated mortalities and sixth among all causes of cancer-related deaths across the world, with malignant tumors. In accordance with the statistics, about 906,000 recently diagnosed patients and 830,000 primary liver cancer death cases were reported 1. HCC increases mortality by approximately 2-3% per year compared with the reduction in mortality from other common tumors, including lung and breast cancer 2. Based on prior epidemiological information, alcohol consumption and hepatitis B/C virus (HBV/HCV) infection are major reasons for HCC. Nonalcoholic fatty liver disease (NAFLD) has been increasingly determined to become a risk factor for HCC over the past five years and is diagnosed in around 1/4 of the world's adults 3, 4. This illustrates the significantly increased risk of NAFLD on the incidence of HCC and the need to analyze the NAFLD-HCC prognosis.

NAFLD indicates steatosis in over 5% of the liver cells after the exclusion of hepatic steatosis caused by heavy alcohol consumption or other toxic substances and drugs 3, 5-7. Recently, MAFLD is redefined as a fatty liver disease by 30 international experts from 22 countries in 2020 5. MAFLD can be diagnosed on the basis of liver steatosis, obesity, prediabetes, hypertension, dyslipidemia, type 2 diabetes mellitus (T2DM), or evidence of metabolic dysregulation 5, 8. Defining diagnosis-related criteria help to identify individuals with metabolic hepatopathy. In addition, it is possible to confirm the MAFLD diagnosis in people of normal weight. As shown in a recent study, a liver biopsy was carried out on 1000 patients with MAFLD. The study suggested that hepatic steatosis severity was approximately identical in patients with a BMI <23 kg/m^2^ and a BMI >25 kg/m^2^ 9. Therefore, metabolic health is more vital than is reflected in the definition. Additionally, MAFLD is significantly associated with 10 types of cancer, including liver cancer 10, 11.

Surgery for liver cancer remains a key approach for obtaining long-term survival of liver cancer patients 12, 13. However, the recurrence rate of HCC after liver cancer resection is high. Recently, a systematic review of large sample data demonstrated that the 5-year overall survival (OS) rates of patients with intermediate or advanced HCC were 42% and 33%, respectively 14, 15. Thus, the relationship between the clinical etiology and HCC prognosis after radical resection of liver cancer needs to be studied in depth. Furthermore, tumor staging and treatment measures are critical factors influencing the survival of HCC patients. Additionally, the effect of MAFLD on HCC prognosis based on cancer and clinical features is poorly understood. Therefore, the current work concentrated on comparing the functions of MAFLD in the survival of HCC patients following curative resection.

## Patients and Methods

### Study type and subjects

**Figure 1:**
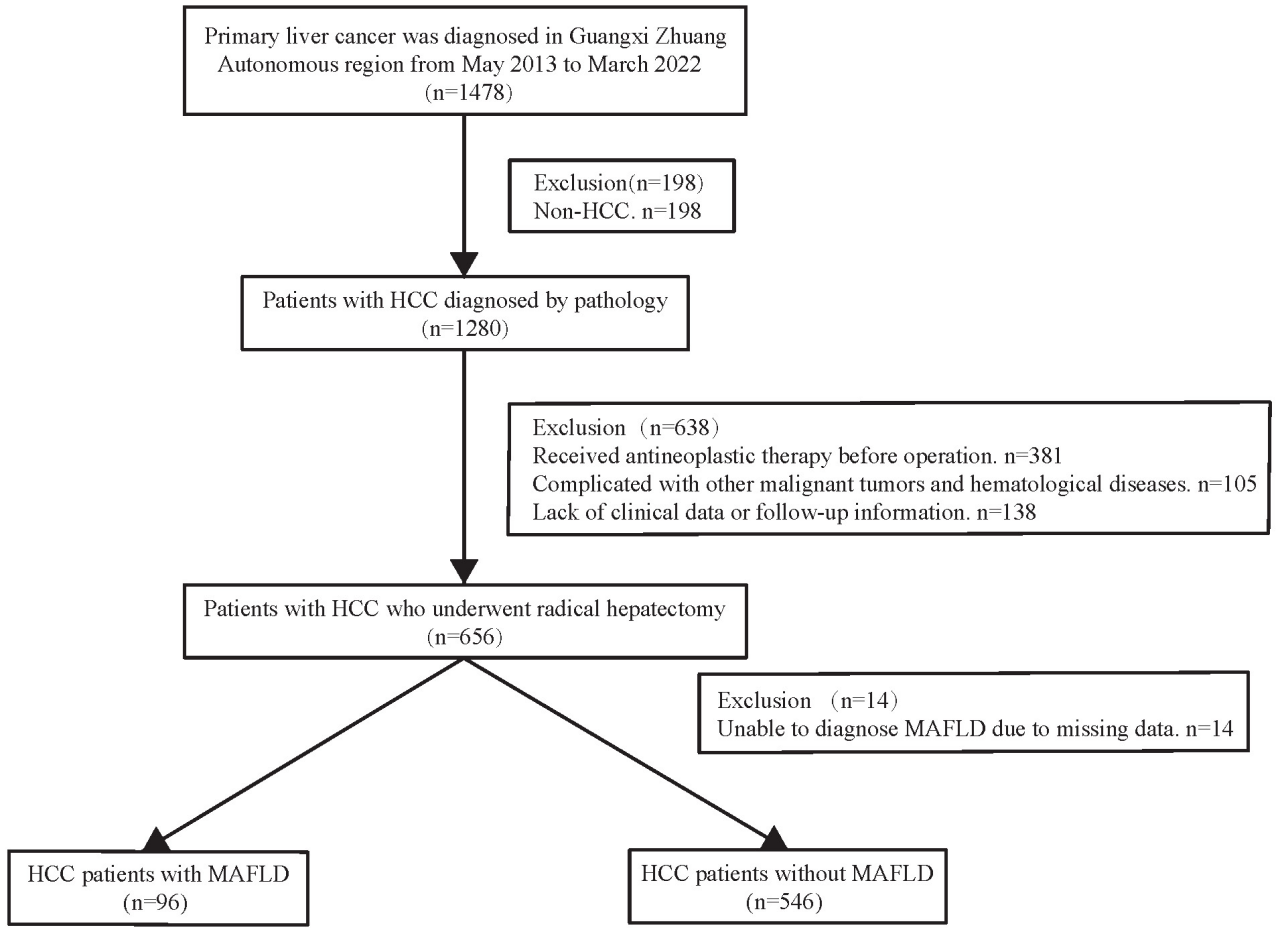
Flow chart for the selection of the study population. Abbreviations: HCC: hepatocellular carcinoma; MAFLD: metabolic dysfunction-associated fatty liver disease.

The present work had a retrospective cohort design. Consecutive patients with HCC diagnosed by pathology between May 2013 and March 2022 were treated with radical resection, following the Chinese guidelines for the diagnosis and treatment of HCC. Liver cancer cases were identified using a big data platform and health management platform from the People's Hospital of Guangxi Zhuang Autonomous Region. We used the present active health management platform to conduct a retrospective cohort study (Registration site: http://www.chictr.org.cn/index.aspx; registration number: ChiCTR2200062446). In brief, an active health management platform used an advanced medical data management system to manage patients, and connected and indexed all diagnostic and treatment records at the hospital. It contained outpatient, inpatient, and physical examination data concerning patient diagnosis. Additionally, treatment data, test reports, examination reports, electronic medical records, and other medical data related to outpatient, inpatient, and physical examinations were also recorded. All medical information can be accessed from this platform, and when a patient arrives at the clinic, the information is automatically integrated into the platform. The following are the inclusion criteria for this study: Patients who met the indications for hepatectomy underwent radical hepatectomy and were histopathologically confirmed to have HCC were contained in the study. Subjects with the following characteristics will be excluded: 1) Those with incomplete clinical data. 2) Individuals diagnosed with hepatobiliary cell carcinoma, mixed hepatocyte-cholangiocarcinoma, or combined with other system malignant tumors (such as lung cancer, colorectal cancer, etc.). 3) Patients with severe cardiac and pulmonary organ dysfunction. 4) Patients with severe infection. 5) Individuals who have not undergone preoperative transcatheter arterial chemoembolization (TACE) or radiofrequency ablation (RFA) for the first time. 6) Patients who are unable to complete the follow-up. The flowchart for patient selection is presented in Figure 1. All operations were performed according to the 1975 Helsinki Declaration. The patients did not provide written informed consent due to the retrospective nature of the present study. The approval of the current work was obtained by the institutional committee of the People's Hospital of Guangxi Zhuang Autonomous Region.

### Definition

Based on the consensus reached by international experts, MAFLD is, diagnosed based on hepatic steatosis (through imaging, liver biopsy, or by using scores and blood biomarkers), combined with one or more of the listed conditions including obesity or overweight, T2DM, or metabolic disorder (those with at least two risk factors for metabolic abnormalities: hypertension, plasma triglyceride ≥ 1.70 mmol/L) 5. The plasma high density lipoprotein-cholesterol (HDL-C) of <1.3 mmol/L and <1.0 mmol/L for women and men, respectively, prediabetes (fasting blood glucose of 5.6-6.9 mmol/L, plasma high sensitivity C-reactive protein (CRP) levels > 2 mg/L, and glycosylated hemoglobin A1c (Hb-A1c) of 5.7%-6.4% are the criteria for defining metabolic risk factors 5. Hypertension condition is determined as diastolic blood pressure (DBP) ≥ 90 mmHg, systolic blood pressure (SBP) ≥140 mmHg or the use of hypertensive medication. Subjects with glycosylated hemoglobin ≥ 6.5, fasting blood glucose (FBG) level ≥7.0 mmol/l, or diabetes was determined as prediabetes. Dyslipidemia-diagnostic code plus drug or laboratory value (LDL cholesterol > 100 mg/dL or triglyceride > 150 mg/dL). Radical resection was defined as no tumor thrombus in the great vein and bile duct, no adjacent organ invasion, and a liver cutting edge ≥ 1cm from the tumor boundary; if the cutting edge was < 1cm, the resection margin of the liver tissue was negative, and no tumor focus was found by imaging examination 1-2 months after the operation 16, 17. Using imaging or histopathological reports, liver steatosis and cirrhosis were identified directly. OS was regarded as the duration between randomization and all-cause mortality. RFS represents the duration from randomization to disease relapse or all-cause mortality.

### Data collection and outcomes

Using the hospital big data and health management platforms, this study obtained the following patients related data: age, sex, body mass index (BMI), Hypertension, T2DM, liver cirrhosis, tumor number, tumor size, Child-Pugh liver function grade, Barcelona clinical liver cancer (BCLC) stage, macrovascular invasion, microvascular invasion, alanine aminotransferase (ALT), plasma triglyceride (TC), MAFLD, total bilirubin (TB) and Triglycerides.

The principal outcome was OS between hepatectomy and all-cause mortality or the final follow-up. None of the patients in this cohort received a living transplant. The secondary endpoints were RFS, perioperative mortality, and morbidity. This study utilized telephone calls, hospital electronic medical records, or outpatient follow-up, with the last follow-up period up to June 30, 2022. The non-recurrence survival period was determined as the period between hepatectomy and disease relapse or all-cause mortality. This study evaluated HCC relapse according to postoperative imaging data (B-ultrasound, CT, and MRI). HCC recurrence is defined as a new lesion after radical resection that fully meets the diagnostic criteria for HCC 18, 19.

### Statistical analysis

Data were explored through SPSS 18.0 (BM Corp, Armonk, NY, USA) and R software (version 3.4.1, The R Project for Statistical Computing). Continuous data were represented by median and interquartile ranges (IQRs), while comparisons among groups were conducted with the Kruskal-Wallis or Mann-Whitney U tests as appropriate. Categorical data were represented by totals and frequencies. In addition, the chi-square test was adopted for comparison. Kaplan-Meier (KM) analysis was conducted with the aim of analyzing OS and RFS, which were compared with the use of the log-rank test. Factors showing significant association with survival (OS and RFS) were identified using multivariate Cox regression to evaluate hazard ratios (HR) and 95% confidence intervals (95% CI) after adjustment for possible confounding factors. Propensity score matching (PSM) analysis with a nearest-neighbor 1:1 matching scheme and a caliper size of 0.05 was used for sensitivity analysis. A *P*-value <0.05 was regarded to be of statistical significance.

## Results

### Basic demographic and clinical features of cases

From May 2013 to March 2022, 1478 patients were diagnosed with primary liver cancer. Finally, 642 patients were enrolled in the current analysis, containing 96 patients diagnosed with MAFLD. The mean age of the enrolled patients was 53 (44-62) years, and 18.1% were female. Compared with men, women were older (58.0 vs. 52.0 years old, *P*<0.05) and had high blood pressure (47.4% vs. 36.7%,* P*<0.05). However, liver synthesis function (measured by total bilirubin and alanine aminotransferase), dyslipidemia, and overweight/obesity in female patients with HCC were better than those in male patients. In addition, microvascular infiltration in females markedly decreased compared with males (29.3% vs. 44.5%; *P*<0.05). However, there were no statistically significant differences in type 2 diabetes, Child-Pugh grade, BCLC grade, macrovascular invasion rate, and tumor size and diameter between women and men (Table 1).

The patients were classified into two subgroups according to MAFLD diagnosis: MAFLD (15.0%) and non-MAFLD (546) groups. This study compared the clinical features of MAFLD and non-MAFLD groups (Table 2). It was shown that the MAFLD group had higher proportions of type 2 diabetes (18.8%) and hypertension (54.2%) than the non-MALFD group (8.6% and 31.9%, respectively;* P*<0.05). The median BMI in the MALFD group (25.6) increased in relative to the non-MAFLD group (21.6) (*P*<0.05). The MALFD group had increased measured triglyceride value relative to the non-MAFLD group (1.2 vs. 1.9 mmol/L; *P*<0.05).

Moreover, the macrovascular infiltration rate in the non-MAFLD group was notably higher than that in the MAFLD group (12.8 vs. 3.1; *P*<0.05). The other basic parameters were comparable between groups (*P*>0.05). In the male and female subgroup analysis, the same results were obtained by comparing the two groups.

### RFS and OS in HCC cases

**Figure 2:**
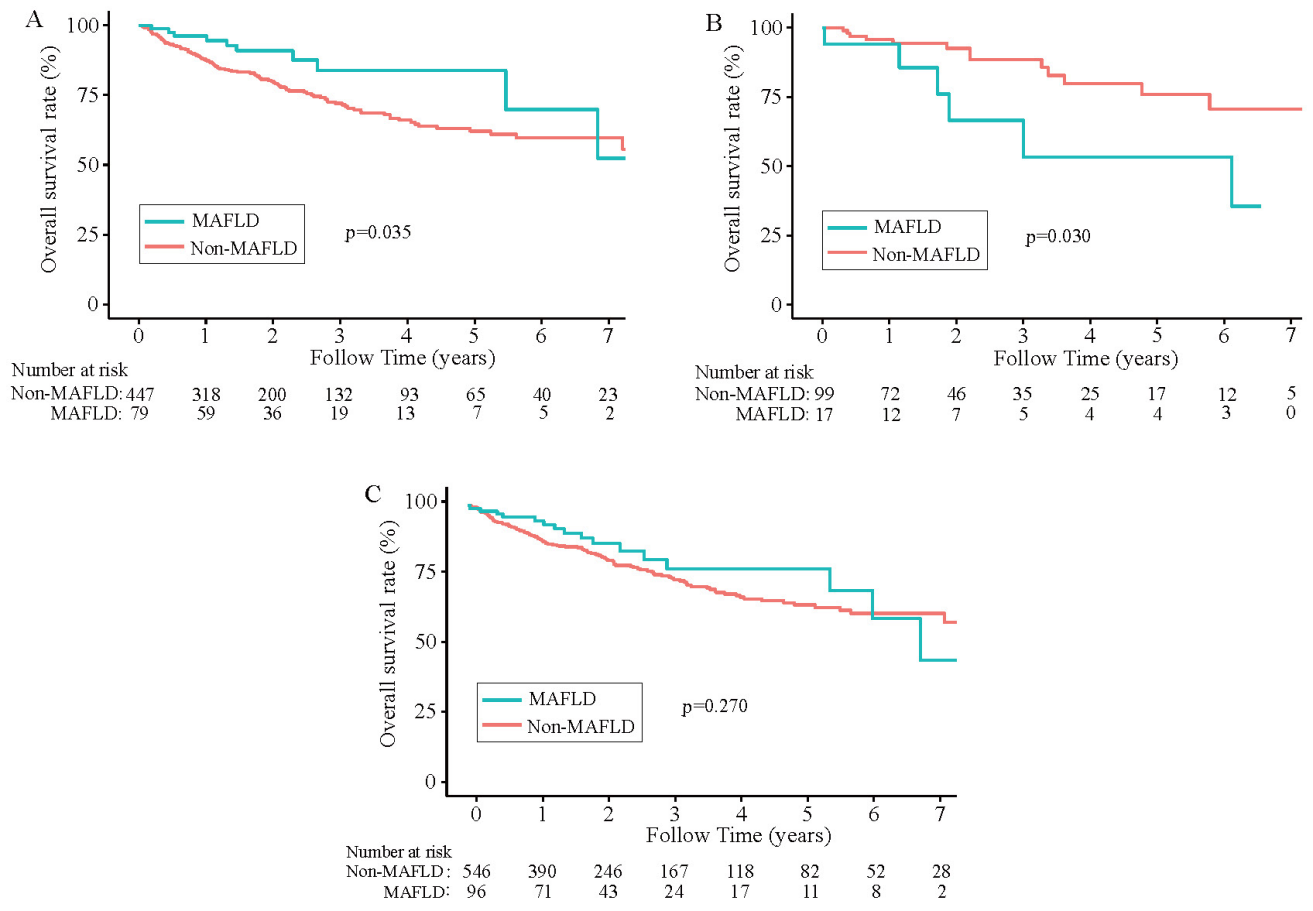
Kaplan-Meier analysis of overall survival for each counterpart. (A) Survival rate of men group (MAFLD vs Non-MAFLD, *P*=0.035); (B) Survival rate of women group (MAFLD vs Non-MAFLD, *P*=0.030); (C) Survival rate of all patients (MAFLD vs Non-MAFLD, *P*=0.270). Abbreviations: MAFLD, metabolic dysfunction-associated fatty liver disease.

**Figure 3:**
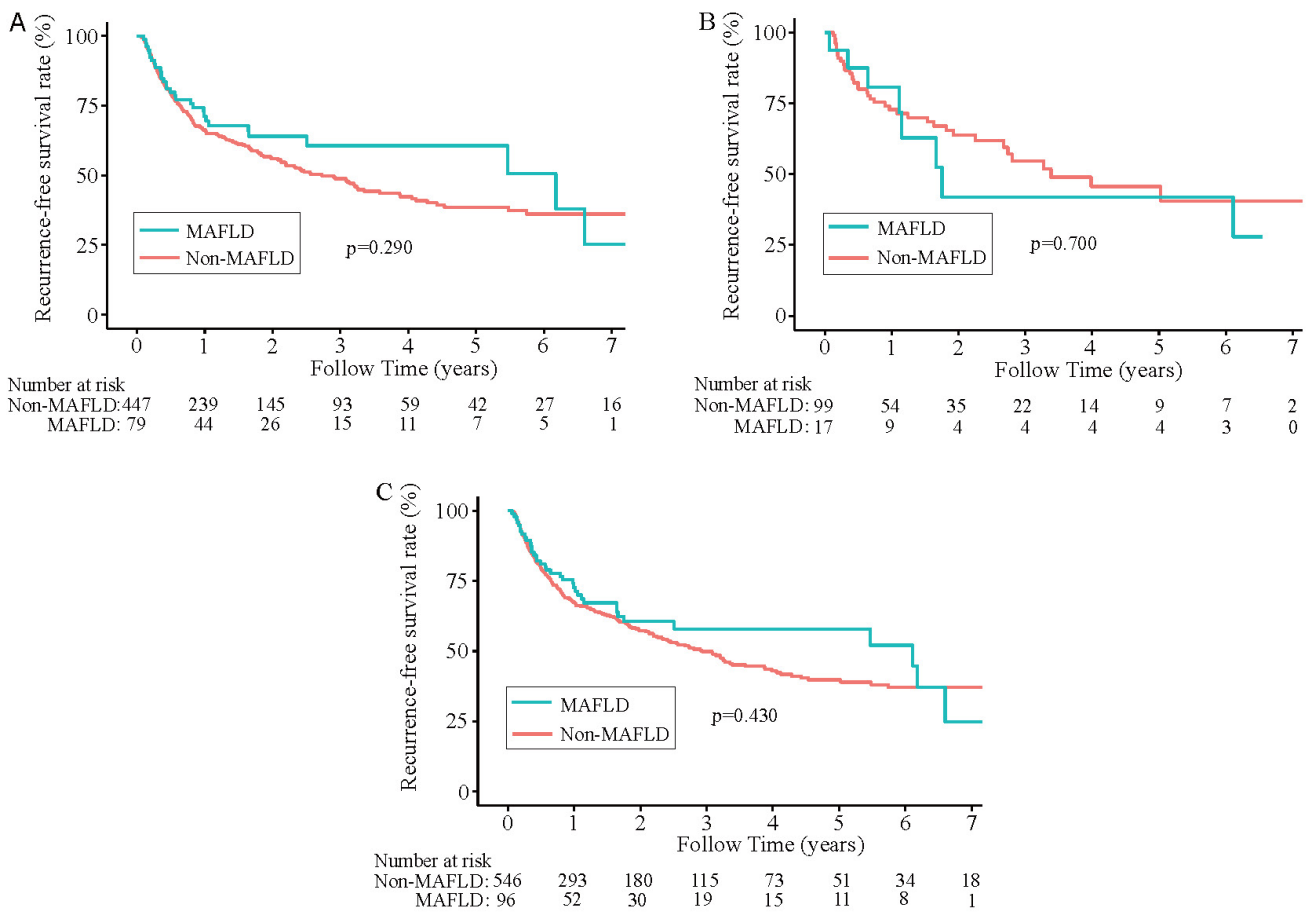
Kaplan-Meier analysis of recurrence-free survival for each counterpart. (A) Recurrence-free survival of men group (MAFLD vs Non-MAFLD, *P*=0.290); (B) Recurrence-free survival of women group (MAFLD vs Non-MAFLD, *P*=0.700); (C) Recurrence-free survival of all patients (MAFLD vs Non-MAFLD, *P*=0.430). Abbreviations: MAFLD, metabolic dysfunction-associated fatty liver disease.

During a median 1.8-year follow-up period [IQR (0.92-3.47) years], 142 (22.12%) of the 642 patients died. In the total population (n =642), the overall survival rates for MAFLD patients at 1, 3, and 5 years were 94.45%, 80.69%, and 77.33%, respectively and in the non-MAFLD group, were 89.06%, 74.88%, and 64.51%, respectively, while the RFS rates for MAFLD patients at 1, 3, and 5 years were 72.68%, 57.92%, and 52.13%, whereas 67.47%, 49.87%, and 39.78%, respectively for non-MAFLD cases. The OS and RFS rates were comparable between the two groups (*P*> 0.05). The KM results demonstrated comparable OS and RFS in both groups for the overall population (*P*>0.05) (Figure 2C, Figure 3C). However, in the male population, the survival rate of the MAFLD group elevated in relative to the non-MAFLD group (*P*<0.05) (Figure 2A). By contrast, in the female population, the survival rate of the MAFLD group decreased compared to the non-MAFLD group (*P*<0.05) (Figure 2B). Moreover, RFS was not statistically significant. among MAFLD and non-MAFLD cases (*P*>0.05) (Figure 3).

### Univariate as well as multivariate regression on RFS and OS-related factors in HCC patients

Tables 3 and 4 summarize the univariate and multivariate analyses of mortality and RFS on the basis of the gender of the patients. MAFLD was risk factor to mortality (HR = 5.177,95%CI1.475-18.193) in female patients, but not statistically significant in male patients and RFS rate.

### Sensitivity analysis using PSM

To minimize the possible confounding bias, we performed PSM in MAFLD group and non-MAFLD group, the characteristics of the two group after PSM was showed in Table S1. Kaplan-Meier analysis performed in the after PSM data showed that the relationship between MAFLD and the OS and RFS in patients with hepatocellular carcinoma after radical resection was consistent to that prior to PSM, which validated our results (Figure S1, Figure S2).

## Discussion

We used a large database from the People's Hospital of Guangxi Zhuang Autonomous Region in order to explore how MAFLD affects patient prognosis after radical resection of liver cancer in a well-matched cohort. In the current observational study, it was found that MAFLD significantly increased mortality in female HCC patients but had no significant effect on RFS and male death. MAFLD increased overall survival and relapse-free survival, but the difference was not of statistical significance.

In the current work, 15% of the HCC cases satisfied the diagnostic criteria for MAFLD, similar to other cohort studies 20. Of the MAFLD-associated HCC patients, 64.6% did not have cirrhosis, suggesting that patients with NAFLD or MAFLD may develop MAFLD and NAFLD-associated HCCs without cirrhosis 21, 22, which highlights the importance of monitoring MAFLD-associated HCC among patients with cirrhotic. Surprisingly, the OS and RFS of MAFLD-HCC cases were superior to those of non-MAFLD-HCC cases in this study, but the difference was not of statistical significance (*P*>0.05). Similar observations were made in a recent study that retrospectively re-analyzed 6882 HCC patients enrolled consecutively in 23 liver cancer centers in Italy from 2002 to 2019 using diagnostic criteria for MAFLD. The study reported that median overall survival (23.8 months) among HCC patients without MAFLD was lower than that of HCC patients with single-cause MAFLD (28.1 months) and HCC patients with mixed causes of MAFLD (27.1 months) 23. Additionally, individuals diagnosed with MAFLD typically exhibit a higher BMI. Conversely, lower BMI values are often associated with conditions such as malnutrition 24. In recent studies, it has been discovered that metabolic factors such as MAFLD 25, 26, obesity 27, and diabetes 28, 29 do not have an effect on the mortality of HCC cases, and MAFLD has been found to increase the surgical rate and liver failure rate after hepatectomy and have a positive impact on long-term prognosis 30, with no significant difference in gender. In this present study, we found that the effect of MAFLD in the prognosis in patients with HCC after radical resection was significantly different between men and women.

According to our results, despite a higher male predominance, the prevalence of diabetes and hypertension in women was still higher than that in men. The World Health Organization estimates that diabetes is more frequently associated with global deaths among women than among men (3.1% vs. 2.3%) 31. The correlation between metabolic syndrome and tumor mortality across South Korea showed that hypertension and blood pressure were obvious risk factors for cancer-associated mortality in women 32. A Japanese cohort study reported results similar to those of our study. During the 18.5-year follow-up period, 473 men and 297 women died due to cancer, and metabolic syndrome showed positive relation to cancer-associated death among females; the opposite was true for men 33. In addition, our data were supported by a cohort study conducted in Canada. The study suggested that metabolic syndrome increases mortality risk among males and females (including 331 cancer cases); however, females were at a higher risk than males 34. Similarly, a recent cohort study conducted in Switzerland suggested that MAFLD leads to a gradually elevated HCC prevalence in women 35. In recent years, a large-scale study in the USA observed that MAFLD cases were related to a 17% 36 higher risk of all-cause death in a median 23-year follow-up period. Based on several prior studies, it can be concluded that metabolic syndrome is strongly related to a higher mortality risk among females than in males 28, 37, 38. The MAFLD definition suggests that metabolic dysfunction plays a crucial role in disease prognosis 5, 8. The above-mentioned studies support our findings that females with MAFLD developing hepatoma are associated with increased mortality compared with males. The relationship between MAFLD and the heightened risk of cancer death remains unclear. However, it may be attributed to factors such as obesity, insulin resistance, and the insulin-like growth factor (IGF) system 39. Obesity can trigger inflammation, which, in turn, can lead to insulin resistance and increase the risk of cancer death 40-42. Insulin can also stimulate the production of IGF-1, which promotes tumor growth 43. This present study found that MAFLD heightens mortality rates in women with HCC, but not in men. BMI is a reliable indicator of overall obesity, and the link between visceral adipose tissue (VAT) and metabolic risk factors is stronger in women than in men 44, 45. Moreover, women tend to release more excess free fatty acids than men, which heightens the risk of the triglyceride/FFA cycle and, in turn, increases the risk of obesity-mediated cancer 46, 47. Consequently, further experiments are needed to analyze the possible heterogeneities in MAFLD and HCC prognosis according to gender.

However, the current work still has the following limitations. First, the data set was obtained from a single institution. Second, this is a retrospective cohort study. Third, the study contained only patients undergoing surgery. Consequently, it is unclear whether our findings are consistent with those of patients receiving other treatments. Furthermore, the sample size was moderate, and the proportion of women was lower than that in other similar studies. In addition, the correlation between MAFLD and HCC survival has not been completely understood because the MAFLD case number examined was relatively small and there was a potential selection bias. Therefore, it is necessary to carry out further careful research with larger sample sizes. Despite the above-mentioned limitations, we believe that the present study offers new and interesting information about gender differences in prognostic effects in patients with MAFLD-related HCC from a retrospective cohort.

In summary, MAFLD can improve the mortality rate of women undergoing radical resection for liver cancer, which independently estimate poor prognosis and is not related to recurrence-free survival.

## Supplementary Material

Supplementary figures and table.

## Figures and Tables

**Table 1:** Clinicopathological characteristics of HCC patients.

Variables	Overall	Men	Women	*P*-value
	n=642	(526, 81.9%)	(116, 18.1%)	
Age, year	53.0 (44.0-62.0)	52.0 (44.0-61.0)	58.0 (48.0-65.0)	0.004
Body mass index, kg/m^2^	23.4 (21.3-25.6)	23.5 (21.5-25.7)	22.5 (20.2-24.9)	0.004
Triglycerides, mmol/L	1.0 (0.8-1.3)	1.0 (0.8-1.3)	1.0 (0.7-1.2)	0.198
Alanine aminotransferase, U/L	33.0 (23.0-52.0)	35.0 (24.0-54.0)	26.0 (17.0-36.5)	<0.001
Total bilirubin, mmol/L	3.2 (2.3-4.5)	3.3 (2.4-4.6)	2.8 (1.9-3.8)	<0.001
Hypertension, n (%)	248 (38.6%)	193 (36.7%)	55 (47.4%)	0.032
Type 2 diabetes mellitus, n (%)	65 (10.1%)	52 (9.9%)	13 (11.2%)	0.669
Dyslipidemia, n (%)	214 (33.3%)	194 (36.9%)	20 (17.2%)	<0.001
Child-Pugh grade				0.181
A	584 (91.0%)	480 (91.3%)	104 (89.7%)	
B	58 (8.7%)	46 (8.6%)	12(9.5%)	
BCLC, stage				0.203
0/A	284 (44.2%)	54 (46.6%)	230 (43.7%)	
B	31 (4.8%)	4 (3.4%)	27 (5.1%)	
C	318 (49.5%)	57 (49.1%)	261 (49.6%)	
D	9 (1.4%)	1 (0.9%)	8 (1.5%)	
Liver cirrhosis, n (%)	248 (38.6%)	209 (39.7%)	39 (33.6%)	0.221
MAFLD, n (%)	96 (15.0%)	79 (15.0%)	17 (14.7%)	0.921
Tumor size, cm	5.0 (3.0-7.7)	5.0 (3.2-7.6)	5.0 (2.8-7.8)	0.879
Tumor number, n (%)				0.559
Single	542 (84.4%)	100 (86.2%)	442 (84.0%)	
Multiple	100 (15.6%)	16 (13.8%)	84 (16.0%)	
Macrovascular invasion, n (%)	73 (11.4%)	61 (11.6%)	12 (10.3%)	0.701
Microvascular invasion, n (%)	268 (41.7%	234 (44.5%)	34 (29.3%)	0.003

Data are mean ± standard deviation, median (IQR) or N (%). Abbreviations: BCLC, Barcelona Clinic Liver Cancer; MAFLD, metabolic dysfunction-associated fatty liver disease.

**Table 2:** Baseline demographic and clinical characteristics of patients with MAFLD or non-MAFLD.

Variables	All patients		*P*-value	Men		*P*-value	Women		*P*-value
	MAFLD	non-MAFLD		MAFLD	non-MAFLD		MAFLD	non-MAFLD	
	(n =96)	(n =546)		(n =79)	(n =447)		(n =17)	(n =99)	
Age, year	55.5 (45.0-64.0)	53.0 (44.0-62.0)	0.146	55.0 (44.5-61.5)	52.0 (43.5-61.0)	0.183	60.0 (51.0-66.0)	57.0 (48.0-64.0)	0.423
BMI, kg/m^2^	25.6 (24.0-27.7)	22.9 (21.0-24.9)	<0.001	25.7 (24.1-27.8)	23.1 (21.2-25.0)	<0.001	25.3 (23.5-26.9)	22.1 (19.9-24.0)	<0.001
Triglycerides, mmol/L	1.2 (1.0-1.6)	0.9 (0.7-1.2)	<0.001	1.2 (1.0-1.6)	0.9 (0.7-1.2)	<0.001	1.1 (0.8-1.4)	1.0 (0.7-1.2)	0.224
ALT, U/L	37.0 (23.5-56.5)	33.0 (23.0-50.0)	0.181	37.0 (24.0-58.0)	34.0 (24.0-53.0)	0.469	35.0 (19.0-50.0)	25.0 (17.0-35.0)	0.139
Total bilirubin, mmol/L	13.0 (10.2-17.2)	13.6 (10.3-18.8)	0.250	13.0 (10.5-17.1)	14.2 (10.6-19.4)	0.130	12.3 (9.7-17.1)	11.9 (9.3-14.5)	0.737
Albumin, g/L	38.7 (36.1-41.0)	37.9 (34.9-40.7)	0.131	38.4 (35.8-40.9)	38.0 (35.0-40.8)	0.436	39.4 (36.9-41.3)	37.3 (34.8-40.0)	0.055
Hypertension, n (%)	52 (54.2%)	196 (35.9%)	<0.001	40 (50.6%)	153 (34.2%)	0.005	12 (70.6%)	43 (43.4%)	0.038
T2DM, n (%)	18 (18.8%)	47 (8.6%)	0.002	14 (17.7%)	38 (8.5%)	0.011	4 (23.5%)	9 (9.1%)	0.081
Dyslipidemia, n (%)	35 (36.5%)	179 (32.8%)	0.481	35 (44.3%)	159 (35.6%)	0.138	0 (0.0%)	20 (20.2%)	0.042
Liver cirrhosis, n (%)	34 (35.4%)	214 (39.2%)	0.483	29 (36.7%)	180 (40.3%)	0.551	5 (29.4%)	34 (34.3%)	0.691
Child-Pugh grade, n (%)			0.518			0.410			0.835
A	89 (92.7%)	495 (90.7%)		74 (93.7%)	406 (90.8%)		15 (88.2%)	89 (89.9%)	
B	7 (7.3%)	51 (9.3%)		5 (6.3%)	41 (9.2%)		2 (11.8%)	10 (10.1%)	
BCLC stage, n (%)			0.107			0.144			0.219
0/A	37 (38.5%)	247 (45.2%)		29 (36.7%)	201 (45.0%)		8 (47.1%)	46 (46.5%)	
B	8 (8.3%)	23 (4.2%)		6 (7.6%)	21 (4.7%)		2 (11.8%)	2 (2.0%)	
C	48 (50.0%)	270 (49.5%)		41 (51.9%)	220 (49.2%)		7 (41.2%)	50 (50.5%)	
D	3 (3.1%)	6 (1.1%)		3 (3.8%)	5 (1.1%)		0 (0.0%)	1 (1.0%)	
Tumor size, cm	4.7 (3.0-6.6)	5.0 (3.1-7.7)	0.237	4.5 (2.8-6.6)	5.1 (3.2-7.6)	0.129	5.3 (4.1-6.7)	4.9 (2.6-7.8)	0.809
Tumor number, n (%)			0.124			0.260			0.208
Single	76 (79.2%)	466 (85.3%)		63 (79.7%)	379 (84.8%)		13 (76.5%)	87 (87.9%)	
Multiple	20 (20.8%)	80 (14.7%)		16 (20.3%)	68 (15.2%)		4 (23.5%)	12 (12.1%)	
Macrovascular invasion, n (%)	3 (3.1%)	70 (12.8%)	0.006	3 (3.8%)	58 (13.0%)	0.019	0 (0.0%)	12 (12.1%)	0.130
Microvascular invasion, n (%)	33 (34.4%)	235 (43.0%)	0.112	32 (40.5%)	202 (45.2%)	0.440	1 (5.9%)	33 (33.3%)	0.022

Data are mean ± standard deviation, median (IQR) or N (%). Abbreviations: ALT, Alanine aminotransferase; T2DM, type 2 diabetes mellitus; BMI, body mass index; BCLC, Barcelona Clinic Liver Cancer; MAFLD, metabolic dysfunction-associated fatty liver disease.

**Table 3:** Univariate and multivariable analyses of mortality according to sex.

Variables	Women				Men			
	Univariable analysis		Multivariable analysis		Univariable analysis		Multivariable analysis	
	HR (95% CI)	*P*-value	HR (95% CI)	*P*-value	HR (95% CI)	*P*-value	HR (95% CI)	*P*-value
Age, year	1.01 (0.98, 1.05)	0.514	1.020 (0.979, 1.062)	0.343	0.98 (0.96, 1.00)	0.013	0.984 (0.967, 1.000)	0.052
ALT, U/L	1.00 (0.99, 1.01)	0.985			1.00 (1.00, 1.00)	0.582		
Albumin, g/L	0.88 (0.81, 0.97)	0.008	0.892 (0.775, 1.027)	0.111	0.98 (0.94, 1.02)	0.350	0.983 (0.940, 1.028)	0.450
Hypertension								
No	1				1.000			
Yes	1.87 (0.75, 4.68)	0.180			0.84 (0.58, 1.23)	0.374		
BMI, kg/m^2^	0.94 (0.81, 1.10)	0.455			0.91 (0.86, 0.97)	0.003		
T2DM								
No	1				1.000			
Yes	2.07 (0.59, 7.21)	0.255			1.01 (0.56, 1.84)	0.961		
Liver cirrhosis								
No	1				1			
Yes	1.27 (0.48, 3.37)	0.627			0.88 (0.59, 1.30)	0.515		
Child-Pugh grade								
A	1		1		1		1	
B	4.12 (1.33, 12.77)	0.014	2.910 (0.426, 19.891)	0.276	1.35 (0.70, 2.59)	0.368	0.791 (0.359, 1.741)	0.560
BCLC stage								
0/A	1				1			
B	4.24 (0.51, 35.30)	0.181			0.86 (0.31, 2.37)	0.769		
C	2.50 (0.95, 6.61)	0.064			1.32 (0.91, 1.93)	0.143		
D	0.00 (0.00, Inf)	0.999			0.73 (0.10, 5.29)	0.755		
Tumor size, cm	1.14 (1.02, 1.27)	0.019	1.117 (0.945, 1.320)	0.195	1.14 (1.09, 1.19)	<0.0001	1.091 (1.029, 1.158)	0.004
Tumor number								
Single	1		1		1		1	
Multiple	2.85 (0.91, 8.94)	0.073	3.656 (1.006, 13.283)	0.049	1.52 (0.94, 2.44)	0.085	1.505 (0.927, 2.441)	0.098
Microvascular invasion								
No	1		1		1		1	
Yes	2.47 (0.98, 6.23	0.055	2.840 (0.893, 9.031)	0.077	2.63 (1.82, 3.79)	<0.0001	2.042 (1.375, 3.030)	0.0004
Macrovascular invasion								
NO	1		1		1		1	
Yes	1.45(0.33, 6.34)	0.619	1.091 (0.175, 6.802)	0.926	2.62 (1.62, 4.22)	<0.0001	1.145 (0.649, 2.002)	0.640
MAFLD								
No	1		1		1		1	
Yes	2.80 (1.06, 7.39)	0.038	5.177 (1.475, 18.193)	0.010	0.51 (0.26, 0.97)	0.039	0.569 (0.292, 1.089)	0.088

Abbreviations: ALT, Alanine aminotransferase; BMI, body mass index; T2DM, type 2 diabetes mellitus; BCLC, Barcelona Clinic Liver Cancer; MAFLD, metabolic dysfunction-associated fatty liver disease.

**Table 4:** Univariate and multivariable analyses of recurrence-free survival according to sex.

Variables	Women				Men			
	Univariable analysis		Multivariable analysis		Univariable analysis		Multivariable analysis	
	HR (95% CI)	*P*-value	HR (95% CI)	*P*-value	HR (95% CI)	*P*-value	HR (95% CI)	*P*-value
Age, year	0.99 (0.97, 1.01)	0.408	0.989(0.965-1.012)	0.3431	0.98 (0.97, 0.99)	0.004	0.988(0.976, 0.999)	0.042
ALT, U/L	1.00 (0.99, 1.00)	0.331			1.00 (1.00, 1.00)	0.935		
Albumin, g/L	0.98 (0.91, 1.04)	0.456	0.974(0.895-1.061)	0.5454	0.97 (0.95, 1.00)	0.040	0.986(0.954,1.020)	0.412
Hypertension								
No	1				1			
Yes	1.02 (0.58, 1.82)	0.936			0.75 (0.57, 0.98)	0.037		
BMI, kg/m^2^	1.03 (0.95, 1.12)	0.463			0.99 (0.95, 1.04)	0.793		
T2DM								
No	1				1			
Yes	1.22 (0.48, 3.09)	0.681			0.90 (0.58, 1.38)	0.624		
Liver cirrhosis								
No	1				1			
Yes	1.32 (0.72, 2.43)	0.369			0.98 (0.75, 1.28)	0.880		
Child-Pugh grade								
A	1				1			
B	1.12 (0.40, 3.16)	0.825	0.953(0.256-3.551)	0.9428	1.71 (1.13, 2.59)	0.012	0.931(0.550,1.575)	0.790
BCLC stage								
0A	1				1			
B	2.11 (0.48, 9.23)	0.320			1.78 (1.05, 3.02)	0.032		
C	1.95 (1.05, 3.62)	0.034			1.02 (0.78, 1.33)	0.903		
D	0.00 (0.00, Inf)	0.998			0.58 (0.14, 2.36)	0.450		
Tumor size, cm	1.09 (1.02, 1.17)	0.013	1.094(0.988,1.212)	0.0833	1.13 (1.09, 1.16)	<0.0001	1.097(1.050,1.147)	<0.0001
Tumor number								
Single	1				1			
Multiple	1.64 (0.76, 3.54)	0.203	1.921(0.808,4.567)	0.1393	1.67 (1.20, 2.32)	0.002	1.510(1.080,2.113)	0.0161
Microvascular invasion								
No	1				1			
Yes	2.49 (1.39, 4.46)	0.002	2.416(1.212,4.820)	0.0123	2.18 (1.68, 2.83)	<0.0001	1.777(1.345,2.349)	<0.0001
Macrovascular invasion								
NO	1				1			
Yes	2.12 (0.94, 4.74)	0.069	1.148(0.437,3.017)	0.7793	2.16 (1.48, 3.14)	<0.0001	1.067(0.678,1.678)	0.780
MAFLD								
No	1				1			
Yes	1.16 (0.54, 2.50)	0.698	1.736(0.720-4.183)	0.219	0.82 (0.56, 1.19)	0.295	0.991(0.674, 1.459)	0.965

Abbreviations: ALT, Alanine aminotransferase; BMI, body mass index; T2DM, type 2 diabetes mellitus; BCLC, Barcelona Clinic Liver Cancer; MAFLD, metabolic dysfunction-associated fatty liver disease.
