# Supplementary material for: Sex Differences in the Impact of Metabolic Dysfunction-associated Fatty Liver Disease on the of Patients with Hepatocellular Carcinoma After Radical Resection
Source: J Cancer. 2023 Apr 17;14(7):1107–16. doi: 10.7150/jca.83779 (PMC10197934; doi:10.7150/jca.83779)
Supplement: Supplementary file 1 — Supplementary figures and table. [file jcav14p1107s1.pdf]

Table S 1
Baseline demographic and clinical characteristics of patients with MAFLD or non-MAFLD after PSM

Variables	All patients		*P*-value	Men		*P*-value	Women		*P*-value
	MAFLD (n =93)	non-MAFLD (n =93)		MAFLD (n =77)	non-MAFLD (n =77)		MAFLD (n =15)	non-MAFLD (n =15)	
Age, year	55.0 (45.0-64.0)	54.0 (46.0-62.0)	0.631	55.0 (44.0-62.0)	52.0 (40.0-60.0	0.166	60.0 (48.0-64.5)	57.0 (53.5-63.0)	0.884
BMI, kg/m^2	25.5 (23.9-27.5)	24.2 (22.2-26.6)	0.008	25.6 (24.0-27.5)	24.5 (22.5-27.2)	0.044	24.0 (23.5-26.3)	22.7 (21.5-25.6)	0.198
Triglycerides, mmol/L	1.2 (1.0-1.5)	1.0 (0.8-1.3)	0.003	1.2 (1.0-1.6)	0.9 (0.7-1.2)	<0.001	1.1 (0.8-1.4)	1.0 (1.0-1.2)	0.624
ALT, U/L	37.0 (22.0-58.0)	33.0 (23.0-51.0)	0.343	37.0 (24.0-60.0)	33.0 (22.0-55.0)	0.453	35.0 (18.0-54.0)	20.0 (14.0-27.5)	0.051
Total bilirubin, mmol/L	13.0 (10.2-17.4)	13.6 (11.0-19.5)	0.253	13.2 (10.4-17.4)	13.5 (10.4-18.8)	0.343	12.3 (9.9-16.0)	10.7 (8.6-16.1)	0.693
Albumin, g/L	38.6 (35.9-41.0)	38.0 (35.2-41.3)	0.567	38.4 (35.7-41.0)	38.5 (35.7-41.5)	0.993	39.4 (36.8-41.3)	40.9 (36.5-41.9)	0.787
Hypertension, n (%)	50 (53.8%)	36 (38.7%)	0.056	39 (50.6%)	35 (45.5%)	0.629	10 (66.7%)	10 (66.7%)	0.999
T2DM, n (%)	16 (17.2%)	13 (14%)	0.686	13 (16.9%)	8 (10.4%)	0.348	2 (13.3%)	2 (13.3%)	0.999
Dyslipidemia, n (%)	34 (36.6%)	32 (34.4%)	0.878	34 (44.2%)	32 (41.6%)	0.871	0 (0)	3 (20%)	0.224
Liver cirrhosis, n (%)	32 (34.4%)	38 (40.9)	0.449	27 (35.1%)	35 (45.5%)	0.250	4 (26.7%)	6 (40%)	0.699
Child-Pugh grade, n (%)									
A	86 (92.5%)	85 (91.4%)	0.999	72 (93.5%)	69 (89.6%)	0.562	14 (93.3%)	14 (93.3%)	0.999
B	7 (7.5%)	8 (8.6%)		5 (6.5%)	8 (10.4%)		1 (6.7%)	1 (6.7%)	
BCLC stage, n (%)			0.229			0.231			0.341
0/A	36 (38.7%)	43 (46.2%)		28 (36.4%)	36 (46.8%)		8 (53.3%)	9 (60%)	
B	8 (8.6%)	5 (5.4%)		6 (7.8%)	5 (6.5%)		2 (13.3%)	0 (0%)	
C	46 (49.5%)	45 (48.4%)		40 (51.9%)	36 (46.8%)		5 (33.3%)	6 (40%)	
D	3 (3.2%)	0 (0)		3 (3.9%)	0 (0)				
Tumor size, cm	4.7 (3.0-6.7)	4.5 (2.9-7.3)	0.894	4.5 (3.0-6.6)	4.7 (3.4-7.0)	0.471	5.3 (4.2-7.8)	3.2 (2.0-7.4)	0.165
Tumor number, n (%)			0.250			0.680			0.327
Single	73 (78.5%)	80 (86%)		61 (79.2%)	64 (83.1%)		11 (73.3%)	14 (93.3%)	
Multiple	20 (21.5%)	13 (14%)		16 (20.8%)	13 (16.9%)		4 (26.7%)	1 (6.7%)	
Macrovascular invasion, n (%)	3 (3.2%)	6 (6.5%)	0.494	3 (3.9%)	11 (14.3%)	0.050	0 (0)	1 (6.7%)	0.999
Microvascular invasion, n (%)	32 (34.4%)	33 (35.5%)	0.999	31 (40.3%)	36 (46.8%)	0.516	1 (6.7%)	3 (20%)	0.591

Data are mean ± standard deviation, median (IQR) or N (%).
Abbreviations: ALT, Alanine aminotransferase; T2DM, type 2 diabetes mellitus; BMI, body mass index; BCLC, Barcelona Clinic Liver Cancer; MAFLD, metabolic dysfunction-associated fatty liver disease.

Figure S 1

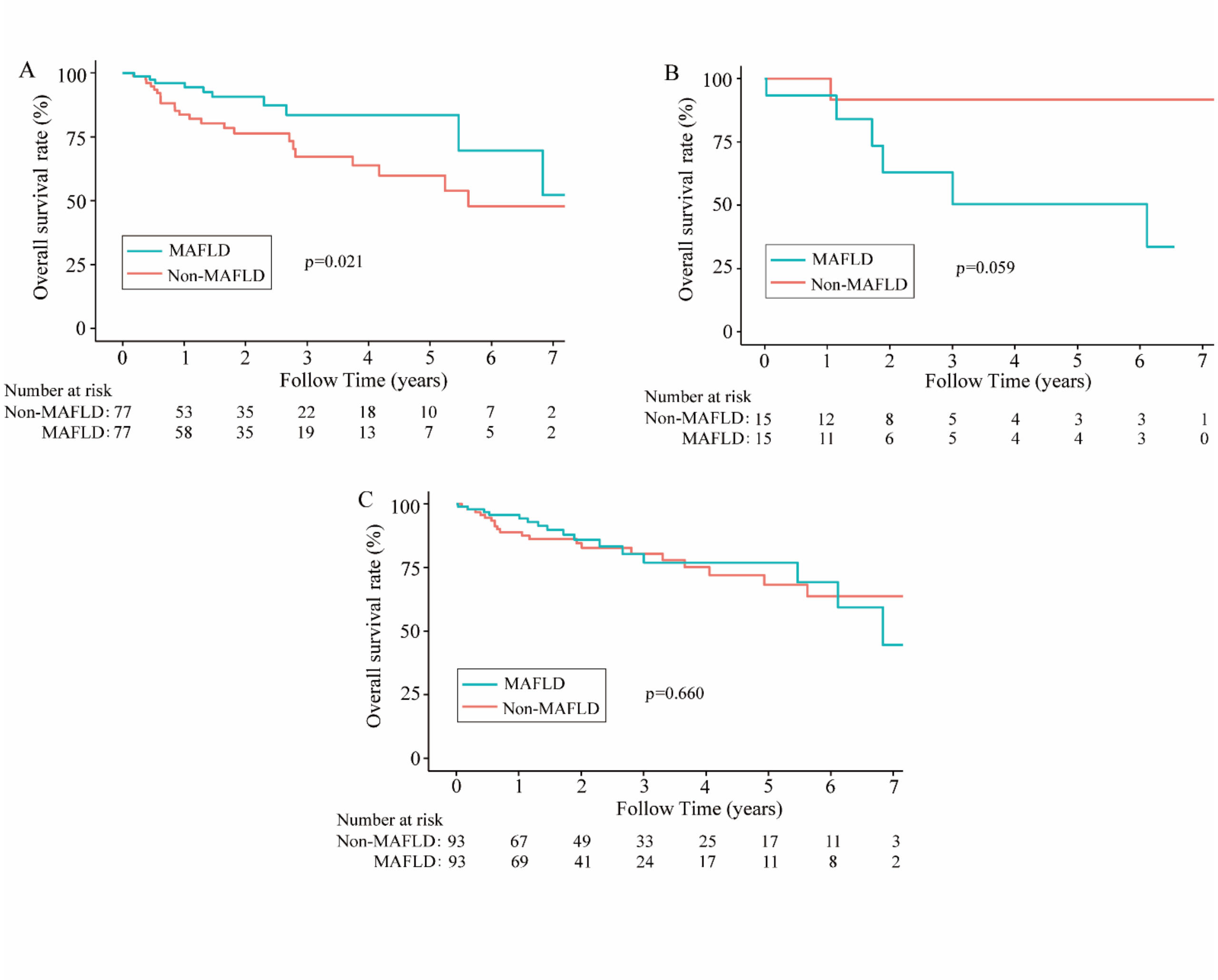

Figure S 1. Kaplan-Meier analysis performed in the after PSM data. (A) Survival rate of men group (MAFLD vs Non-MAFLD, *P*=0.021); (B) Survival rate of women group (MAFLD vs Non-MAFLD， *P*=0.059); (C) Survival rate of all patients (MAFLD vs Non-MAFLD, *P*=0.660). Abbreviations: MAFLD, metabolic dysfunction-associated fatty liver disease.

Figure S 2

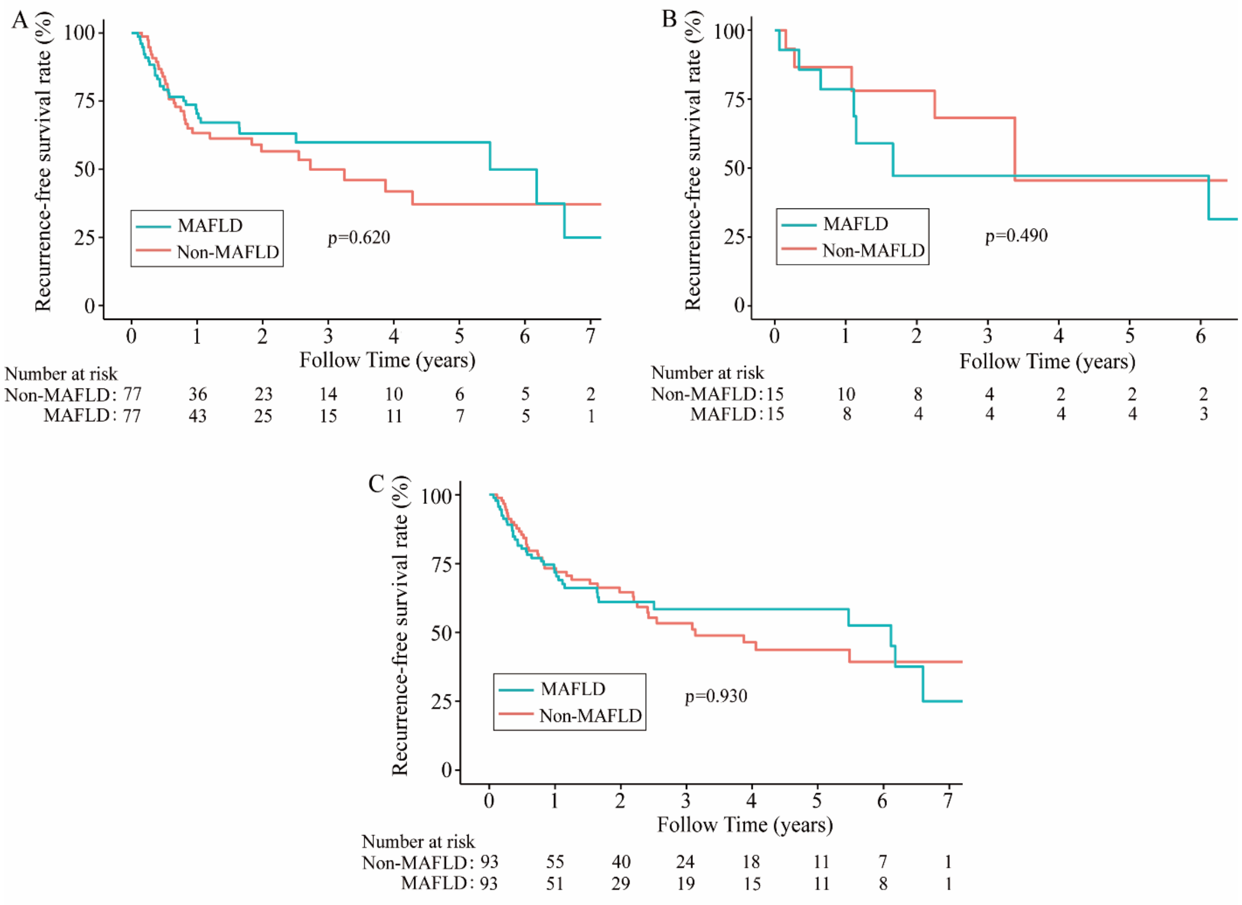

Figure S 2. Kaplan-Meier analysis performed in the after PSM data. (A) Recurrence-free survival of men group (MAFLD vs Non-MAFLD, P=0.620); (B) Recurrence-free survival of women group (MAFLD vs Non-MAFLD， P=0.490); (C) Recurrence-free survival of all patients (MAFLD vs Non-MAFLD, P=0.930).

Abbreviations: MAFLD, metabolic dysfunction-associated fatty liver disease.
